# Brain Sex Differences Related to Gender Identity Development: Genes or Hormones?

**DOI:** 10.3390/ijms21062123

**Published:** 2020-03-19

**Authors:** Jiska Ristori, Carlotta Cocchetti, Alessia Romani, Francesca Mazzoli, Linda Vignozzi, Mario Maggi, Alessandra Daphne Fisher

**Affiliations:** 1 Andrology, Women’s Endocrinology and Gender Incongruence, Careggi University Hospital, Viale Pieraccini 6, 50139 Florence, Italy; jiskaristori@gmail.com (J.R.); carlotta.cocchetti@gmail.com (C.C.); alessiaromani@hotmail.it (A.R.); francesca.mazzoli@stud.unifi.it (F.M.); linda.vignozzi@unifi.it (L.V.); 2 Endocrinology, Careggi University Hospital, Viale Pieraccini 6, 50139 Florence, Italy; m.maggi@dfc.unifi.it

**Keywords:** genes, hormones, sexual differentiation, brain sexual dimorphism, gender identity

## Abstract

The complex process of sexual differentiation is known to be influenced by biological and environmental determinants. The present review has the aim of summarizing the most relevant studies on the biological basis of sexual development, and in particular, it focuses on the impact of sex hormones and genetic background on the development of sexual differentiation and gender identity. The authors conducted a search of published studies on Medline (from January 1948 to December 2019). The evidence suggests that the sexual dimorphic brain could be the anatomical substrate of psychosexual development, on which gonadal hormones may have a shaping role during prenatal and pubertal periods. Additionally, according to several heritability studies, genetic components may have a role, but a promising candidate gene has not been identified. Even though growing evidence underlines the primary role of biological factors on psychosexual development, further studies are necessary to better explain their complex interactions.

## 1. Introduction

The process of sexual differentiation refers to the development of differences between males and females, which are widely observed in nature and also concern humans [1,2,3,4]. One of the most sexually dimorphic human traits is gender identity [5], defined as the inner sense of self as a female, a male or as an alternative gender, different from the male and female ones [6]. In *cisgender* individuals, gender identity develops in line with the assigned gender at birth and is stable throughout life [7]. On the other hand, *transgender* individuals may persistently or transiently identify with a different gender different from the one assigned at birth. According to the Diagnostic and Statistical Manual of Mental Disorders 5^th^ version (DSM 5) [6], we refer to Gender Dysphoria when the incongruence between the experienced/expressed gender and the assigned one leads to clinically significant psychological distress and impairment in the main areas of functioning [6]. In some cases, this distress may lead to the desire for a social and/or somatic transition through a gender affirming hormonal treatment and surgery [6,8,9,10]. 

Considering this complex frame and also its clinical implications, large attention has been dedicated to understand the origins of the sexual differentiation process. There is a great debate in literature on the factors related to nature or nurture involved in the sexual differentiation of the brain. However, it is well established that biology plays a major role. In fact, in the last few years research has focused mainly on neuroanatomy and sexual dimorphism of the brain, exploring the influence and shaping role of several genes and sex hormones [4]. In particular, the sexual dimorphic brain is considered the anatomical substrate of psychosexual development, on which genes and gonadal hormones may have a shaping effect [11]. Growing evidence shows that prenatal and pubertal sex hormones permanently affect human behaviour and heritability studies have demonstrated a role of genetic components. 

Indeed, cismen and ciswomen present anatomical differences in the total brain volume, as well as in several sex-dimorphic structures. In particular, the total brain volume is bigger in cismen, and in transgender men similar volumes to the assigned gender at birth were found [12,13,14,15]. However, the total intracranial volume in transwomen resulted to be in between male and female controls [12]. Furthermore, sex differences have been observed in cortical thickness that is higher in ciswomen compared to cismen in several regions [16,17]. Studies conducted on transgender individuals reported signs of feminisation in cortical thickness of transwomen, while no sign of masculinisation was found in transmen [18,19]. 

Another sexual dimorphic area seems to be represented by the amygdala and the hippocampus. Indeed, the amygdala is larger in cismen and with a higher density of androgen than oestrogen receptors, whereas portions of the hippocampus are larger in women, with a higher density of oestrogen than androgen receptors (AR) [20,21]. 

These differences raise the question whether cross-gender identifications reflect the brain anatomy and/or function. For this reason, a growing literature is focusing, with both post-mortem and in vivo neuroimaging studies, on structural and functional differences between transgender and cisgender individuals in several areas of the brain, especially in those that show sexual dimorphism. 

Regarding grey matter, the main sexually dimorphic areas associated with the development of gender identity are represented by the central subdivision of the bed nucleus of the stria terminalis (BNST) and the third interstitial nucleus of the anterior hypothalamus (INAH3). Post-mortem studies reported that the BNST is smaller and with low somatostatin neurons in ciswomen and transwomen compared with cismen [22]. Regarding the INAH-3, which is involved in sexual and maternal behaviours and in the secretion of gonadotropins [23], one study reported this area to be smaller in transwomen than in cismen and to have less neurons [24]. However, the role of BNST and INAH-3 in the determination of sexual differentiation remains unclear because of the small size of the samples and because part of the subjects enrolled had received hormonal treatment previously. Additionally, the majority of individuals with gender dysphoria report cross gender identity since childhood, while sex differences in BNST do not appear before puberty [25].

Sex differences in the brain also emerged when focusing on white matter characteristics [13,26,27,28]. Indeed, white matter microstructure was evaluated via diffusion tensor imaging (DTI) that measures the functional anisotropy (FA) of white matter. This presents differently in men and women, with men usually having a greater FA value than women [13,26]. Studies conducted on transgender individuals described patterns of white matter microstructure to be more in line with the perceived gender (rather than the biological sex) [13,26,27,28]. However, to date, these limited data do not allow to provide a reliable conclusion. 

Furthermore, since cognitive abilities are notably sexually specific, research has focused on neurofunctional differences assessed trough task-based functional imaging studies. In fact, it is well known that women have better performances in verbal fluency tasks, while men in the visuospatial ones. Considering that, similar studies conducted on transgender individuals may add information about the hypothesis of the possible organisational-activational effect of hormones on the brain [29,30,31,32,33,34]. In a study by Schoning et al. [30], transwomen differed from cismen in brain activation pattern during a mental rotation task. Contrastingly, Soleman et al. [31] found no differences between transgender adolescents and a control group in terms of neuronal activation during verbal fluency tasks. Another study by Junger et al. [32] reported that transwomen showed different neural activation patterns when listening to male versus female voices, showing an intermediate position between the two control groups. 

In conclusion, the existence of brain phenotypes in line with the idea of a brain sexual differentiation seems to be confirmed by the latter reported studies, including both cisgender and transgender individuals. However, the relationship between gender behavioural differences and brain dimorphic areas is still not clear, since such differences may be the result not only of anatomical features but also life experiences [34,35,36,37]. Furthermore, the popular explanation that there is a female and a male brain on the base of gender behavioural differences is not supported by a strong empirical background [11], as, for example, men and women share more similarities than differences [38,39,40,41,42,43]. Furthermore, a great variability in behavioural and psychological aspects is shown between genders [44]. Moreover, the size of the brain differences is usually small [45,46,47]. 

### The Shaping Effect of Sex Hormones and Involved Genes on Brain Sex Differentiation

Human sexual development is a dynamic process regulated and influenced by both genetic and endocrine factors [48,49]. In order to explore the role of gonadal hormone secretion during the prenatal period in sexual differentiation of the brain, several studies on gender identity and sexual orientation have been conducted [11,50,51]. In fact, sex hormones might play organizational and activational effects on the brain and behaviour [51]. Some studies reported sexual and behavioural shifts in female rodents after the administration of testosterone during a critical period for foetal brain organization [52]. According to this theory, while the differentiation of sexual organs happens in the first two months of pregnancy, brain sexual differentiation follows in the last trimester of the pregnancy through permanent organizing effects induced by sex hormones on the developing brain [53,54]. These structures will be activated by sex hormones during puberty. In line with this hypothesis, some authors explain the origin of gender dysphoria as the result of the genital and brain differentiation not being in line. This explanation does not find a complete consent and a more complex interaction should be taken into account. For example, Raznahan et al. [55] speculated that gonadal hormones may maintain or increase basic neuroanatomical differences between sexes in puberty and maybe later on. 

Indeed, the impact of prenatal hormones on gender identity development is still not clear [56]. The effect of prenatal androgen exposition has been explored with studies conducted on *typical population* by using indirect measures, such as finger ratio (i.e., the length of the index finger to the ring finger length [57]), which is higher in females since intrauterine life [58,59,60,61,62,63,64]. Finger ratio might be considered as a marker of prenatal androgen levels, with a lower 2D:4D levels indicating high prenatal testosterone and low oestrogen, and a higher 2D:4D low prenatal testosterone and high oestrogens [65]. However, research on the relationship between finger ratio and gender identity has produced inconsistent results [66,67,68]. Another indirect indicator of prenatal hormones exposure is otoacoustic emission (OAEs)—the weak sound produced by the auditory transduction apparatus of the inner ear. In fact, OAEs present differently in males and female, being weaker in newborn males than in newborn females. Furthermore, these differences persist throughout the lifespan [69]. Transwomen displayed a more female-typical OAE, confirming the hypothesis that they have been exposed to lower levels of androgens during early development compared to control boys [70]. The role of sex hormone exposure in utero is underlined by the observation that prenatal exposure to anticonvulsant—which may interfere with sex hormones metabolism—was associated with the development of gender dysphoria [71]. 

Interesting observations come from studies conducted on *intersex individuals.* This sample represents a unique model to assess if and how sex hormones may interfere with the establishment of sex differences with a particular regard to sexual orientation and gender identity [72,73,74,75,76,77,78,79,80,81,82,83,84,85,86,87,88,89]. Comparing women with congenital adrenal hyperplasia (CAH) to female controls, more cross-gender typical role behaviour and patterns during childhood [72,73,74,75,76] with a preference for typically male toys [72,74,77,78,79,80,81] and playmates [81,88] was reported. Additionally, other studies showed a delay of sexual experiences, lower maternalism and higher prevalence of bi- or homosexual orientation in women with CAH compared to the general female population [80,82]. Concerning gender identity, a few cases of gender dysphoria have been described in this population, leading to the decision of a female to male gender reassignment [83,84,85,86,87]. Although there is strong evidence (40.9%) of more typical male behaviours [73], the majority (95%) of 46,XX CAH individuals were raised as females and developed a female gender identity. However, the reported rate of gender dysphoria and/or male identification in CAH females is 5% [88], exceeding gender dysphoria in the general population (5% vs. 0.002–0.003% in CAH vs. female general population, respectively [6]). In these studies, gender identity was correlated with neither the severity of the CAH condition nor the degree of genital virilization. Subsequently genital appearance at birth does not represent an adequate predictor for gender identity outcomes in these subjects [89]. Regarding the role of the androgen receptor pathway in the development of gender identity, interesting evidence may come from 46, XY individuals with Complete Androgen Insensitivity Syndrome (CAIS). In fact, in CAIS patients, with complete lacking function of the androgen receptor, female gender identity is usually reported indicating that masculinization of gender identity depends of androgen exposure during foetal period [90]. However, two cases of male gender identity in CAIS individuals have been reported in literature [91,92,93], questioning the role of androgen receptor in brain masculinization. Observations from other intersex conditions—such as 5-alpha-reductase-2 deficiency—lead to assume a potential role of pubertal hormones in the development of gender identity. In fact, a study reported a high rate (56–63%) of gender role change from female to male during adolescence and adulthood [94]. However, data on the relationship between the development of a male gender identity and circulating androgens before, during and after gender role change were lacking in the latter study. In addition, masculinization of the brain may occur in 46,XY individuals with cloacal exstrophy assigned to the female sex at birth who underwent orchiectomy in infancy [95]. For this reason, even if other factors such as cultural and environmental background may influence the development of gender identity, evidence from individuals with intersex conditions confirms the critical role of prenatal androgen exposure in sex differentiation of the brain. However, to date, interactions between biological and environmental factors remain still largely unknown.

Additional information on the role played by sex hormones in determining sexually dimorphic brain characteristics may derive from the impact of hormonal treatment in transgender individuals. Literature on this field remains limited, especially with regards to studies with a longitudinal design. 

In transgender individuals, the administration of gender-affirming hormonal treatment may influence anatomical and functional brain characteristics, considering the high density of oestrogen and androgen receptors here expressed [96,97,98]. Indeed, in transwomen oestrogen plus anti-androgen treatment resulted in reducing brain volume and increasing ventricles dimensions [99] and led to a general decrease in cortical thickness [100]. On the other hand, testosterone treatment in transmen determined an increase in total brain and hypothalamus volumes [99], as well as in cortical thickness and cortical-subcortical volumes, specifically the right thalamus [100]. Aside from those changes in grey matter, Rametti et al. [27] described in transmen an increase of FA values in two white matter fascicles a few months after the start of testosterone treatment. The authors hypothesised that testosterone treatment may induce the latter changes through its anabolic and anticatabolic action. In transwomen, the suppression of testosterone levels due to antiandrogens may cause a reduction of grey matter, leading to a decrease in cortical thickness and expansion of ventricles in addition to a putative direct effect of oestrogens [100]. Although the limited number of longitudinal studies does not allow to draw firm conclusions, this evidence again highlights the plasticity of the brain in response to sex hormones even in adulthood. 

Sexual differentiation of the brain and development of gender identity seem to also be affected by genetic factors [2]. Twin studies represent a good model to assess the heritability of a certain trait and provide an important contribution in the definition of genes’ role in the development of gender identity. Indeed, if a trait is more concordant in monozygotic twins compared to dizygotic ones, this provides good evidence that the trait is heritable. In a retrospective study, Bailey et al. [101] reported a heritability pattern for gender non-conformity during childhood in a large sample of adult twins. In addition, Burri et al. [102] showed a small heritability for adult gender identity, analysing a sample of 4426 British female twins by using a non-validated scale. Focusing on a gender dysphoria diagnosis, Coolidge et al. [103] identified a strong heritable component (62% of the variance), even if in this study gender dysphoria symptoms were reported by mothers, possibly interfering with the results. 

In the literature, we can also find some cases of more than one transgender within the same family [104], as well as few twin cases [105,106,107,108]. In support of a role for genetic factors in gender dysphoria development, a review of case studies of twins showed a higher concordance for gender dysphoria in monozygotic twins than dizygotic ones [109]. However, results from these studies may be affected by the role of environmental influences [2]. 

Guided by the role of sex hormones in brain sexual differentiation—as previously reported—several candidate genes have been studied. In particular, research has focused on polymorphisms in steroidogenic enzymes or in steroid receptors, which may lead to different biological activity. 

One of the candidate genes is represented by the *CYP17* gene, which encodes the 17-alpha hydroxylase enzyme. This enzyme converts 17-hydroxypregnenolone to dehydroepiandrosterone and 17-hydroxyprogesterone to androstenedione. In two studies a significant association between transmen and a particular *CYP17* single nucleotide polymorphism was found [110,111]. This polymorphism is associated with elevated serum and plasma levels of oestradiol, progesterone and testosterone. The latter finding is in line with the hypothesis that increased tissue availability of testosterone may interfere with early brain development, fostering the development of a male gender identity. 

Furthermore, research focused on the androgen receptor gene as a potential candidate gene implicated in the development of gender identity and brain sexual dimorphism. Indeed, the complete loss of function of this gene leads to a female gender identity. This gene contains a longer *(CAG)nCAA*-repeat polymorphism which confers a reduced functioning of the receptor, limiting biological activity of testosterone. Accordingly, some studies demonstrated an increased number of trinucleotide CAG repeats in the androgen receptor gene in transwomen [112,113], while others found contrasting results [114,115].

Studies on polymorphism of the *oestrogen receptor beta (ERb*) gene also led to conflicting evidence. Henningson et al. [113] found a significant association between transwomen and a dinucleotide CA polymorphism in the *ERb*. Furthermore, the contribution of this polymorphism was apparently much stronger for subjects carrying relatively few CAG repeats in the androgen receptor [113]. However, this result was not replicated in other studies [112,114]. Fernández et al. [114] found a repeat number in *ERb* gene significantly higher in transmen compared to controls. This result highlights the possibility of a correlation between the size of the polymorphism and receptor functioning, with a higher number of repeats implying a greater transcription activation. In natal females during prenatal period this may lead to an increased defeminization of the brain [111].

More recently, Foreman et al. [116] conducted a study on a large sample of transwomen and control males, evaluating several candidate genes. The authors found a significant association between gender dysphoria and oestrogen receptor alpha (*ERα), SRD5A2 and STS* alleles, as well as *ERα* and *SULT2A1* genotypes. These genetic variants could be functional, influencing oestrogen signalling. In fact, in *SULT2A1*, the genotype associated with gender dysphoria leads to elevated levels of sex hormone binding globulin, inducing a decreased effect of circulating hormones during intrauterine period. In the same way, the *SRD5A2* allele evaluated in this study may lead to a reduction of DHT levels, thus determining a reduction of this potent androgen among transwomen. The authors identified several allele combinations overrepresented in transwomen, mostly involving AR, which may lead to long CAG repeats of the AR.

In conclusion, the evidence from these studies support the idea that brain sexual differentiation and the development of gender identity have a polygenic basis, involving interactions among multiple genes and polymorphism. However, results are in most cases conflicting and the number of genetic studies remains limited. 

## 2. Discussion

The aforementioned studies, although very heterogeneous, provide data supporting the biological bases of the psychosexual development. In particular, post-mortem and in vivo neuroimaging studies strongly suggest the existence of a sexual dimorphic brain, i.e., slight differences in brain anatomy and functioning between the two sexes. It is less clear how such brain structures become the substrate of sex differences in cognition and behaviour. This matter has been mainly investigated through the examination of specific populations, such as subjects with gender incongruence and *intersex* individuals: gender identity is one of the most sex-specific human trait, and many studies show how brain sexually dimorphic structures are often in line with gender identity rather than with sex assigned at birth. Research on this field has reported a possible organizational-activational role of sex hormones: in fact, studies on people with intersexual conditions highlight the role of prenatal and pubertal sex hormones in the determination of gender identity and other sex-specific cognitive traits. This evidence is also supported by the data from studies on hormonal treatment of transgender persons: indeed, a little but promising group of longitudinal studies also demonstrated the brain plasticity in response to cross-sex hormonal treatment in adult life. Anyway, to provide reliable conclusions, more data are needed. In fact, it is important to note that the size of the brain sex differences is really small, and that life experiences could have a deep impact on brain development. Additionally, little is known about the specific biological activity of sex hormones on brain structures: in particular, further studies should examine the role of androgens and oestrogens brain receptors. 

Besides sex hormones, genetic factors are supposed to be the main determinants of brain sexual differentiation: again, the study on allele variations in transsexual individuals allowed to identify several candidate genes, mostly involving sex hormones receptors or steroidogenic enzymes, as possible determinants of sexual differentiation. The results were contrasting, but they may suggest the hypothesis of a polygenic basis of gender identity; in any case, the complex interaction between these genetic factors is far from understood, and that should be the matter of further studies. 

## 3. Conclusions

Prenatal and pubertal sex hormones seem to permanently affect human behaviour and, in addition, heritability studies have demonstrated a role of genetic components. However, a convincing candidate gene has not been identified. Future studies (i.e., genome wide studies) are needed to better clarify the complex interaction between genes, anatomy and hormonal influences on psychosexual development. 

## Abbreviations

DSM 5	Diagnostic and Statistical Manual of Mental Disorders 5^th^ edition
AR	Androgen Receptor
BNST	Bed Nucleus of the Stria Terminalis
INAH3	Interstitial Nucleus Of The Anterior Hypothalamus
DTI	Diffusion Tensor Imaging
FA	Functional Anisotropy
OAE	Otoacoustic Emission
CAH	Congenital Adrenal Hyperplasia
CAIS	Complete Androgen Insensitivity Syndrome
ER	oestrogen receptor
